# The *Phytophthora sojae* Avirulence Locus *Avr3c* Encodes a Multi-Copy RXLR Effector with Sequence Polymorphisms among Pathogen Strains

**DOI:** 10.1371/journal.pone.0005556

**Published:** 2009-05-15

**Authors:** Suomeng Dong, Dinah Qutob, Jennifer Tedman-Jones, Kuflom Kuflu, Yuanchao Wang, Brett M. Tyler, Mark Gijzen

**Affiliations:** 1 Agriculture and Agri-Food Canada, London, Ontario, Canada; 2 Nanjing Agricultural University, Nanjing, China; 3 Virginia Bioinformatics Institute, Virginia Polytechnic Institute and State University, Blacksburg, Virginia, United States of America; University of California, Berkeley, United States of America

## Abstract

Root and stem rot disease of soybean is caused by the oomycete *Phytophthora sojae*. The avirulence (*Avr*) genes of *P. sojae* control race-cultivar compatibility. In this study, we identify the *P. sojae Avr3c* gene and show that it encodes a predicted RXLR effector protein of 220 amino acids. Sequence and transcriptional data were compared for predicted RXLR effectors occurring in the vicinity of *Avr4/6*, as genetic linkage of *Avr3c* and *Avr4/6* was previously suggested. Mapping of DNA markers in a F_2_ population was performed to determine whether selected RXLR effector genes co-segregate with the *Avr3c* phenotype. The results pointed to one RXLR candidate gene as likely to encode *Avr3c*. This was verified by testing selected genes by a co-bombardment assay on soybean plants with *Rps3c*, thus demonstrating functionality and confirming the identity of *Avr3c*. The *Avr3c* gene together with eight other predicted genes are part of a repetitive segment of 33.7 kb. Three near-identical copies of this segment occur in a tandem array. In *P. sojae* strain P6497, two identical copies of *Avr3c* occur within the repeated segments whereas the third copy of this RXLR effector has diverged in sequence. The *Avr3c* gene is expressed during the early stages of infection in all *P. sojae* strains examined. Virulent alleles of *Avr3c* that differ in amino acid sequence were identified in other strains of *P. sojae*. Gain of virulence was acquired through mutation and subsequent sequence exchanges between the two copies of *Avr3c*. The results illustrate the importance of segmental duplications and RXLR effector evolution in the control of race-cultivar compatibility in the *P. sojae* and soybean interaction.

## Introduction

In the interaction between plant pathogens and their hosts, discrete genes control race-cultivar compatibility and disease outcome. Disease resistance resulting from the interplay of pathogen avirulence *(Avr)* genes and host resistance (*R*) genes was first described in the gene-for-gene hypothesis [1]. This type of plant disease resistance is now called effector triggered immunity (ETI) [2]. Typically, plant *R*-genes are part of the innate immune system and encode surveillance proteins that detect pathogen-specific molecules or alterations and thereby activate ETI [3]. Likewise, pathogen effector proteins can be broadly described as secreted proteins that suppress plant defenses and promote disease [4], [5].

The identification of the first *Avr* genes from oomycetes, together with whole genome sequencing projects, revealed a special class of secreted effector proteins that are delivered into host cells [6], [7], [8], [9], [10], [11]. These proteins contain a second targeting motif, downstream from the signal peptide, with the consensus sequence RXLR (Arg-X-Leu-Arg). The RXLR and associated dEER (Asp-Glu-Glu-Arg; with the leading Asp being variable) motifs somehow traffic the pathogen effector protein across the host plasma membrane [12], [13]. The RXLR effectors constitute large super-families of rapidly evolving proteins in all oomycete genomes sequenced to date [14], [15]. Predicted RXLR effector genes in *P. sojae* have been named *Avirulence homologues*, or *Avh* genes.

The central importance of RXLR effectors in determining the outcome of oomycete-plant interactions is becoming increasingly evident. Oomycete *Avr* genes identified and shown to encode RXLR effectors include: *Avr1b*-1, *Avr1a* and *Avr3a* from *P. sojae* [6], [16], *Avr3a*, *Avr4*, and *Avr-blb1* from *Phytophthora infestans* [9], [17], [18]; and *ATR1* and *ATR13* from *Hyaloperonospora arabidopsis* [7], [8]. These findings demonstrate that the RXLR effector family is at the forefront in evolution and adaptation of oomycete plant pathogens towards their hosts. The genes encoding RXLR effectors are subject to unstable selective pressures that shape this rapidly evolving and highly diverse gene family [14], [15], [16]. Once an RXLR effector comes under *R*-gene mediated surveillance and causes ETI, it becomes an *Avr* gene. Evasion of ETI may be accomplished by a variety of mechanisms including amino acid changes, protein truncations, gene deletions, or by transcriptional silencing of the *Avr* gene. Thus, the massive redundancy and the high level of intra- and inter-specific variation of RXLR genes provide a diverse reservoir of effector capability to meet the ever changing selective pressures imposed by plant immune systems.

Soybean *R*-genes that control ETI to *P. sojae* are known as *Rps* (*Resistance to P. sojae*) genes [19]. The aim of the present study was to identify the *Avr3c* gene from *P. sojae* and to determine how this varies among strains with differential virulence on *Rps3c*. Candidate genes for *Avr3c* were selected based upon genetic mapping data, genome sequence assemblies, and the predicted RXLR secretome of *P. sojae*. Functional characterization of the *Avr3c* gene shows this to correspond to an RXLR effector that varies in amino acid sequence among *P. sojae* strains.

## Results

### Gene candidates for *Avr3c* are chosen from predicted RXLR effectors near *Avr4/6*

**Figure 1:**
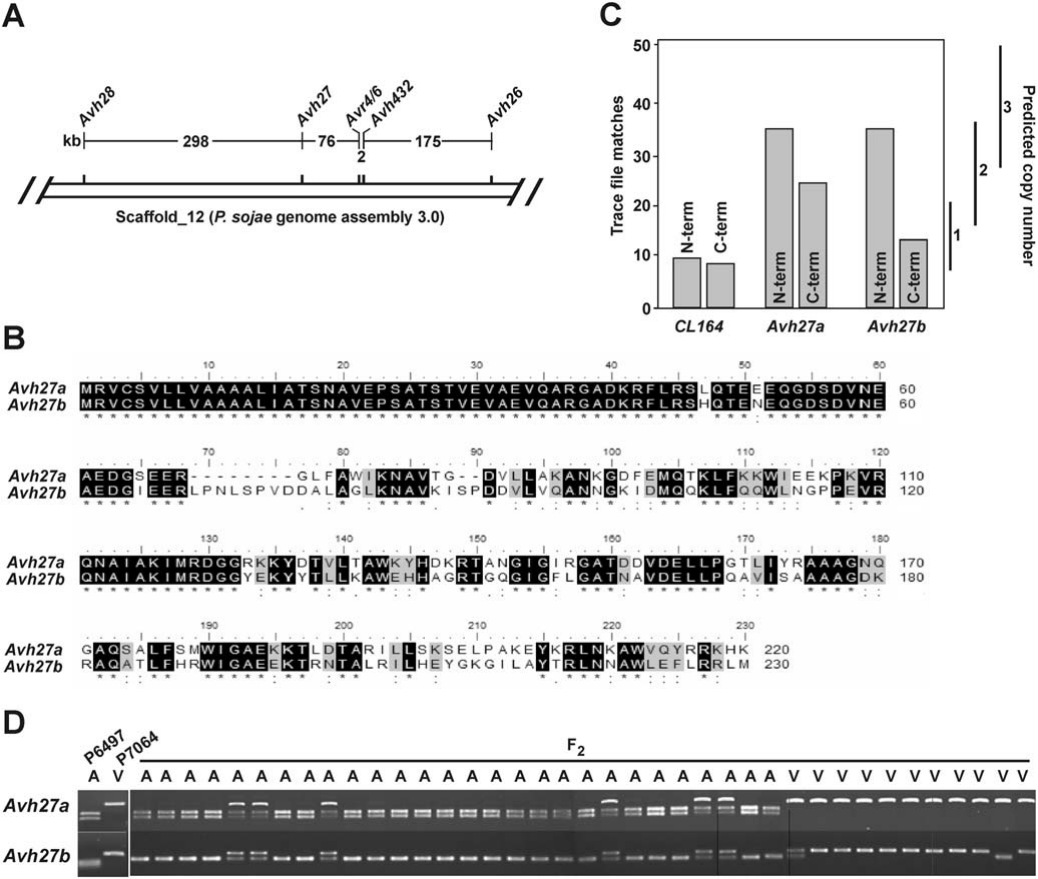
Gene candidates for *Avr3c* are identified in the *Avr4/6* region. (A) Physical map of the Avr4/6 region, showing the position of *Avh* genes in the vicinity. (B) Predicted amino acid sequences for Avh27a and Avh27b. (C) Depth sampling of the *Avh27a* and *Avh27b* genes. Segments of 100 bp from the amino-terminal (N-term) or carboxy-terminal (C-term) end of the open reading frames of each gene were compared to 1,533,511 trace files from whole genome shotgun sequencing of *P. sojae* P6497. The number of sequence matches with an expect (E) value<10^−40^ is indicated on the left axis. The predicted number of matches for a segment with one, two, or three copies per haploid genome is shown to the right of the graph, with the bars indicating the 95% confidence interval for each class. Results from a single copy reference gene, *CL164*, are shown for comparison. (D) Co-segregation of *Avh27a* but not *Avh27b* with *Avr3c* virulence phenotype in F_2_ progeny. A cross of *P. sojae* P6497×P7064 generated the F_2_ progeny. The cultures were scored as virulent (V) or avirulent (A) on *Rps3c* soybean plants. Cleaved amplified polymorphic (CAP) markers, co-dominant and specific for each parental allele of *Avh27a* and *Avh27b*, were scored using genomic DNA from the parents and F_2_ progeny. Shown is a photograph of an ethidium bromide-stained agarose gel of the CAP marker results.

The completion of the *P. sojae* P6497 genome sequence [10], together with the mapping and recent identification of *Avr4/6* [20], [21] provided an opportunity to identify *Avr3c*. Previous genetic mapping work placed *Avr3c* and *Avr4/6* on a single linkage group, separated by a distance 16 cM [22]. The overall average genetic to physical distance in *P. sojae* is approximately 35 kb/cM, but the *Avr4/6* region has a high recombination frequency, estimated at 3 kb/cM [20], [23]. Thus, a 16 cM distance may represent a physical range of 48 to 560 kb, depending upon whether this is calculated using the recombination frequency determined for *Avr4/6* or the genome average. By examining the space around *Avr4/6*, we determined there are at least four additional RXLR effector genes predicted to occur within a 551 kb interval encompassing the gene, as shown in Figure 1A. Thus, *Avh26*, *Avh27*, *Avh28*, and *Avh432* are predicted RXLR effector genes that lie in the vicinity of *Avr4/6* and represent good candidates for *Avr3c*.

Sequence and transcript analysis of each of the candidate genes was performed on *P. sojae* strains P7064 and P7074, to detect any polymorphisms compared to the sequences from *P. sojae* P6497. These *P. sojae* strains differ in virulence characteristics on soybean plants carrying *Rps3c*, *P. sojae* P6497 is avirulent while P7064 and P7074 are virulent. The results are summarized in Table 1. This analysis showed that the *Avh28* and *Avh432* sequences are identical in virulent and avirulent strains of *P. sojae*, and that no transcriptional polymorphisms could be detected. The *Avh26* sequence in P7064 and P7074 differs by a single nucleotide from that in P6497, causing an amino acid change in the predicted protein, but no transcript could be detected for *Avh26* in any of the *P. sojae* strains. In contrast, comparison of the *Avh27* sequence revealed many nucleotide and amino acid differences between virulent and avirulent *P. sojae* strains. Furthermore, *Avh27* was determined to be present in multiple copies in each of the *P. sojae* strains. A pair of closely related sequences, named *Avh27a* and *Avh27b*, was assembled from trace files from whole genome sequence data (Figure 1B). Each of the genes, *Avh27a* and *Avh27b*, differ in nucleotide and predicted amino acid sequence in *P. sojae* strain P7064 compared to P6497. The copy number of each gene was estimated by depth-sampling of trace files, because previous work has shown that this method presents a reliable way to estimate the number of copies of particular RXLR genes in *P. sojae* P6497. Sampling of trace files suggests that *P. sojae* P6497 contains two identical copies of *Avh27a* and a single copy of *Avh27b* (Figure 1C). Analysis of genomic DNA of *P. sojae* P6497 using *Avh27a*-specific primers by real-time PCR resulted in a determination of 2.05±0.404 copies per haploid genome, a value in agreement with the estimate from depth sampling.

**Table 1:** Summary of transcript and sequence analyses of *Avr3c* candidate genes in various *P. sojae* strains.

Strain^a^	*Avh28*		*Avh27*		*Avh432*		*Avh26*	
	mRNA^b^	Sequence^c^	mRNA	Sequence	mRNA	Sequence	mRNA	Sequence
P6497 (A)	(+)	Single copy	(+)	Multi-copy	(−)	Single copy	(−)	Singly copy
P7064 (V)	(+)	Single copy, no polymorphisms	(+)	Multi-copy; numerous non-synonymous SNP	(−)	Single copy, no polymorphisms	(−)	Single copy; single non-synonymous SNP
P7074 (V)	(+)	Single copy, no polymorphisms	(+)	Multi-copy; numerous non-synonymous SNP	(−)	Single copy, no polymorphisms	(−)	Single copy; single non-synonymous SNP

a *P. sojae* strains compared were avirulent (A) or virulent (V) on *Rps3c* plants.

b (+), mRNA detected; (−), mRNA not-detected. Transcriptional analysis was performed by RT-PCR on mRNA isolated from infected tissues, 12 h and 24 h post inoculation.

c Sequence polymorphisms of *P. sojae* strains P7064 and P7074 are in comparison to reference strain P6497; SNP, single nucleotide polymorphism.

### *Avh27a* corresponds to *Avr3c*

**Figure 2:**
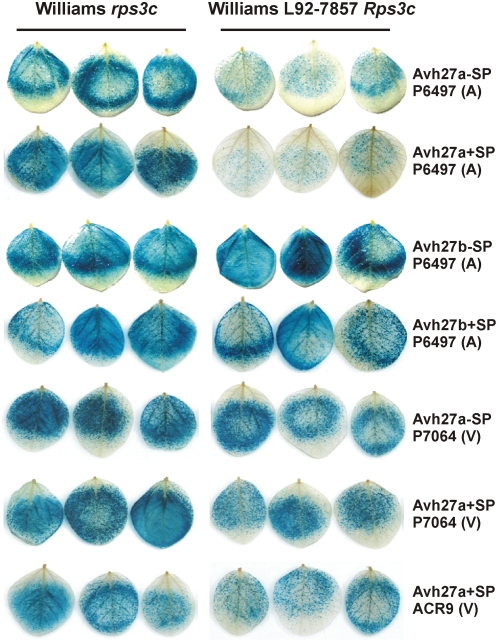
Transformation and transient expression indicates that *Avh27a* is *Avr3c*. Soybean leaves of cultivar Williams (*rps3c*) or Williams L92-7857 (*Rps3c*) were transformed by co-bombardment with plasmids encoding a glucuronidase reporter gene (GUS) and a test construct, using tungsten beads. Expression of each gene, the GUS reporter and the test construct, is driven by the 35S promoter. The various test constructs used in combination with the GUS reporter are indicated on the right. Test constructs with their native signal peptide intact (+SP) or deleted (−SP) are shown. The *P. sojae* strain (P6497, P7064, or ACR9) that provided the source of the test constructs is also indicated. Results from co-bombardment of three separate leaves, for each cultivar and test construct, are shown.

To determine whether *Avh27a* or *Avh27b* cosegregate with *Avr3c*, we developed DNA markers for each of the genes and tested a collection of F_2_ progeny that was additionally scored for virulence on *Rps3c*, as shown in Figure 1D. This analysis demonstrates that *Avh27a* precisely cosegregates with *Avr3c*, whereas recombination between *Avh27b* and *Avr3c* is evident. With all of the results pointing to *Avh27a* as the best candidate for *Avr3c*, we preformed DNA transformation by co-bombardment of soybean leaves, to test whether *Avh27a* interacts with *Rps3c*. Plasmid constructs of *Avh27a* and *Avh27b*, with and without native signal peptide, were bombarded into soybean leaves along with a reporter gene to measure cell viability, as shown in Figure 2. Results indicate that expression of *Avh27a* triggers cell death specifically on plants carrying *Rps3c*, regardless of the presence of the signal peptide. Transformation with *Avh27b* did not result in any differences in reporter gene expression in comparison to controls, indicating that this gene does not trigger cell death on *Rps3c* plants. Thus, *Avh27a* from *P. sojae* P6497 was renamed *Avr3c^P6497^*.

### The *Avr3c* transcript is expressed early during infection

**Figure 3:**
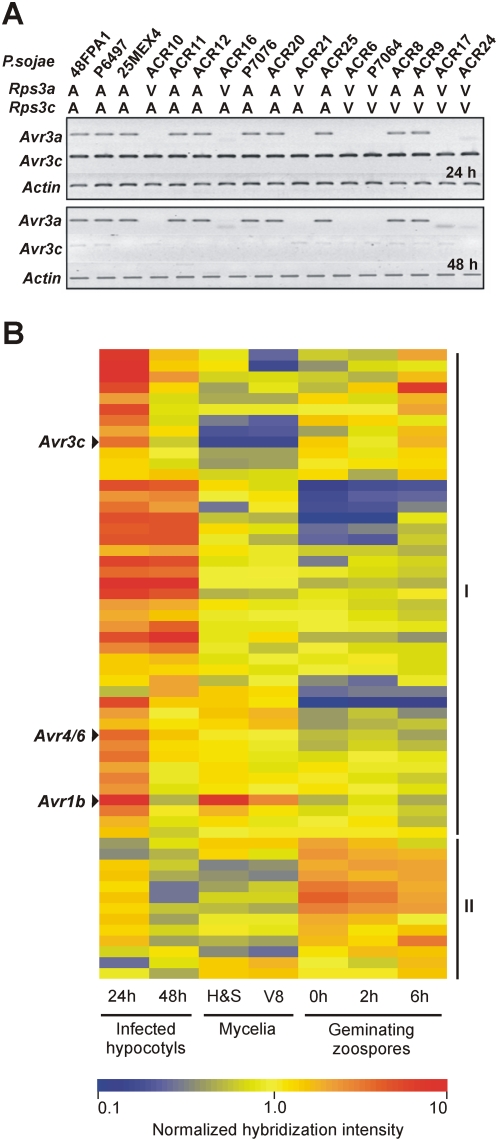
Transcript analysis of RXLR effectors reveals differences in expression. (A) A comparison of *Avr3a* and *Avr3c* expression in 17 different *P. sojae* strains, at 24 h and 48 h after infection of soybean hypocotyls. The strains were scored as virulent (V) or avirulent (A) on *Rps3a* and *Rps3c* soybean plants. Total RNA was extracted from infected plant tissues and provided template for RT-PCR analysis, employing primers specific for *Avr3a*, *Avr3c* and the *P. sojae actin* gene. All infections for RNA isolation were performed on the cultivar Harosoy, a plant that is susceptible to all 17 *P. sojae* strains tested here. Amplification products are shown in this negative image of an ethidium bromide-stained agarose gel. (B) Microarray expression results for the *P. sojae* RXLR effector family. Shown are normalized hybridization intensities for 58 *P. sojae* RXLR effectors. Total RNA extracted from *P. sojae* P6497 and infected plant tissues (cultivar Harosoy) provided template for synthesis of cDNA probes used in the hybridizations. Infected hypocotyls were sampled 24 h and 48 h after inoculation. Mycelia were grown on synthetic media (H&S) or vegetable juice media (V8). Zoospores were sampled at 0 h, 2 h and 6 h after inducing encystment and germination by agitation. The RXLR effectors that correspond to known *Avr* genes are indicated. Cluster analysis of gene expression patterns produced two groups of genes: one group (I) of 45 genes with highest expression during infection, and a second group (II) of 13 genes with highest expression in germinating zoospores.

The expression of *Avr3c* was compared in a collection of *P. sojae* strains to determine whether any difference in transcript levels could be associated with virulence phenotypes. The *Avr3a* gene was also measured for comparison, because transcription of this gene is known to vary among *P. sojae* strains. Results shown in Figure 3A demonstrate that *Avr3c* is expressed in all *P. sojae* strains tested, in contrast to *Avr3a*. For *P. sojae* strains with detectable *Avr3a* transcripts, the expression of this gene differed from *Avr3c*. The expression of *Avr3c* peaked at 24 h after infection and declined rapidly, being scarcely detectable at the 48 h time point, whereas comparable levels of *Avr3a* transcript were present at 24 h and 48 h. Thus, expression of *Avr3a* and *Avr3c* differs markedly. Furthermore, the uniformity of expression of *Avr3c* among the different *P. sojae* strains indicates that virulence is not associated with loss of the *Avr3c* transcript, for any of the strains that were tested.

The expression of *Avr3c* in *P. sojae* P6497 was also compared to predicted RXLR genes represented on a commercially available microarray. Of the 15,820 *P. sojae* target sets on the array, we determined that 107 of these exactly match to one or more of the 385 predicted RXLR effectors from *P. sojae* [14]. Hybridizations were performed using mRNA isolated from germinating zoospores, infected plant tissues (compatible interaction), and *in vitro* grown mycelia. By applying a cut-off filter, we found that only 58 of the 107 array targets displayed hybridization intensities that were above background levels in one or more of the seven treatments. This set of 58 expressed RXLR effectors included *Avr3c*, *Avr1b*-1, and *Avr4/6*. The *Avr3a* gene was not among the 107 targets on the array. Expression patterns of the 58 RXLR effectors were arranged by cluster analysis, as shown in Figure 3B. Two broad groups that displayed contrasting expression patterns emerged from this analysis. The first group of 45 RXLR effectors showed highest relative expression during infection, while the second group contained 13 RXLR effectors that were preferentially expressed in germinating zoospores. Varying patterns of expression within each of the groups was also noted. For example, considering the first group of effectors with highest expression during infection, expression of many of these abruptly declined after the 24 h time point while others continued to be expressed at high levels at 48 h. The expression of *Avr3c*, *Avr1b*-1, and *Avr4/6* reached their highest levels at 24 h after inoculation then declined at 48 h.

### *Avr3c* is embedded in a tandem array

**Figure 4:**
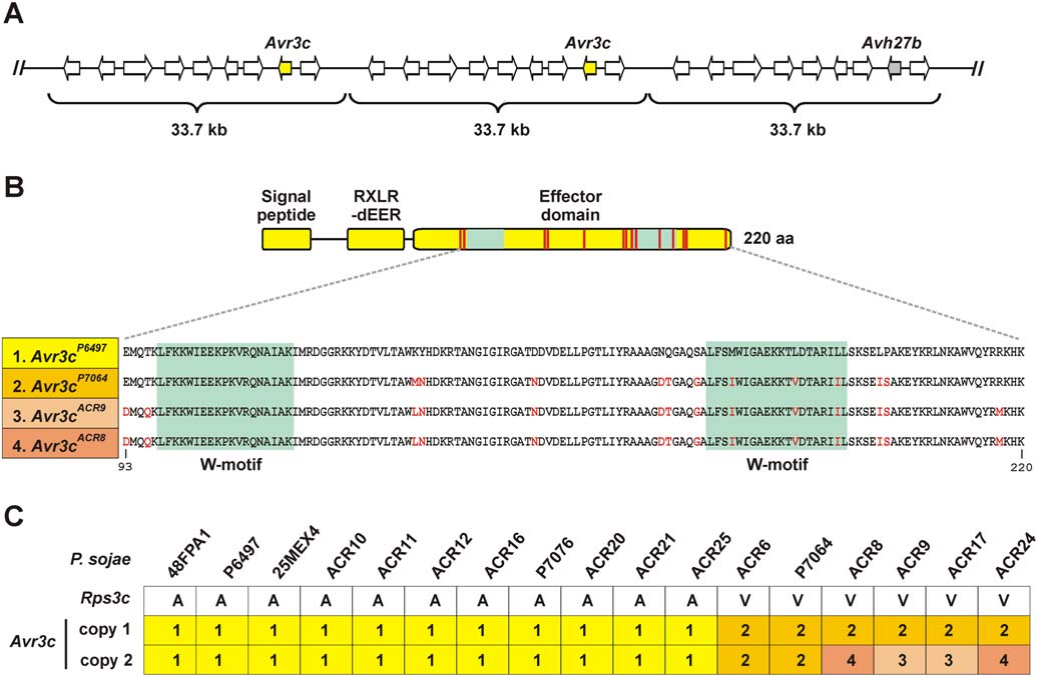
Evolution of the *Avr3c* gene cluster in *P. sojae*. (A) A model for the organization of *Avr3c* in *P. sojae* P6497. The *Avr3c* gene is embedded in a 33.7 kb repetitive segment. The position of two *Avr3c* genes, *Avh27b*, and eight other predicted genes in the repetitive segment are shown; arrow indicates direction of transcription. A list of predicted genes occurring in the replicated segment is provided in the Table S1. (B) The upper portion shows the Avr3c pre-protein, including the position of the signal peptide, RXLR-dEER motif, and the effector domain. The positions of mutations causing amino acid changes to Avr3c are indicated with red bars; green boxes show W-motifs. The lower portion shows the predicted amino acid sequences of a portion of the effector domain of the four *Avr3c* alleles (numbered 1 to 4), encoding three distinct protein products. Variant amino acid residues are shown in red. The *Avr3c^ACR9^* and *Avr3c^ACR8^* alleles differ in only in nucleotide sequence. (C) Haplotype analysis of *Avr3c* gene copies in 17 different *P. sojae* strains. The strains were scored as virulent (V) or avirulent (A) on *Rps3c* soybean plants. The *Avr3c* haplotype was determined by DNA sequence analysis, using genomic DNA template purified from each of the 17 *P. sojae* strains shown in the figure. The two copies of *Avr3c* in each *P. sojae* strain were scored and numbered according to the different alleles shown in part (B).

To determine the organization of the genetic space around *Avr3c* we examined the genome assembly of *P. sojae* P6497, and compared this to results from DNA blot and real-time PCR analyses, and depth-sampling of trace files. Results indicate that *Avr3c* and *Avh27b* are embedded within a 33.7 kb tandem repeat, as shown in Figure 4A. A list of predicted genes occurring in the replicated segment is provided in the Table S1. Each 33.7 kb repeat contains nine predicted genes, including one RXLR effector gene corresponding to *Avh27b* (one copy) or *Avr3c* (two copies). Other *P. sojae* strains examined appear to have a similar structural arrangement. However, comparison of conserved syntenic regions in three additional oomycete species indicates the *Avr3c* interval has diverged substantially in *P. sojae* (Figure S1). The arrangement of many of the flanking genes remained conserved in *P. ramorum*, *P. infestans*, and *Hyaloperonspora arabidopsis*, but these regions do not encode an *Avr3c* ortholog nor any predicted RXLR effectors whatsoever.

### Gain of virulence is caused by amino acid changes in the effector domain of *Avr3c*

Since there were no differences in expression of *Avr3c* among *P. sojae* strains, we sequenced this gene in each strain to determine any allelic differences, and tested whether virulent alleles evaded recognition by *Rps3c*. An alternate allele present in P7064, now called *Avr3c^P7064^*, displayed 11 differences in its deduced amino acid sequence in comparison to the Avr3c^P6497^ protein, as shown in Figure 4B. Two other *Avr3c* alleles were discovered that showed DNA sequence differences compared to *Avr3c^P6497^*. These two alleles, represented by *Avr3c^ACR8^* and *Avr3c^ACR9^*, differ in DNA sequence from each other but the predicted protein products encoded by their ORF are identical (Figure 4B). The Avr3c^ACR8^ and Avr3c^ACR9^ protein products are indistinguishable from each other and nearly identical to Avr3c^P7064^, differing by only four amino acids. Each of the three distinct Avr3c proteins were 220 amino acids in length and contained two predicted W-motifs within the effector domain. The *P. sojae* strain-specific amino acid differences in Avr3c were localized exclusively to the effector domain. In four strains of *P. sojae*, we could isolate two different copies of the *Avr3c* gene (Figure 4C). Since copy number analysis indicated that *Avr3c^P6497^* is present in two identical copies in *P. sojae* P6497, we conclude that the two copies of *Avr3c* have diverged slightly in sequence in the *P. sojae* strains ACR8, ACR9, ACR17, and ACR24. It was important to test each of the *Avr3c* alleles for interaction with *Rps3c* by the co-bombardment assay, because all *P. sojae* strains tested expressed this gene despite their differences in virulence on *Rps3c*-containing plants. These results demonstrate that *Avr3c* alleles from virulent strains of *P. sojae* do not cause cell death on *Rps3c* plants, in contrast to the *Avr3c^P6497^* allele present in P6497 and other avirulent strains (Figure 2). Thus, we conclude that *Avr3c^P6497^* is recognized by *Rps3c* but *Avr3c^P7064^* and *Avr3c^ACR8^* are not.

## Discussion

The identification of oomycete *Avr* genes is now happening at a rapid pace, facilitated by genome sequence data and the discovery of the RXLR host-targeting motif. This is occurring after more than 30 years of intensive research to find Avr factors in *P. sojae* and *P. infestans*. It is remarkable that at least nine different *Avr* genes corresponding to RXLR effectors have been described in three species of oomycete plant pathogens in the last five years [6], [7], [8], [9], [16], [17], [18], [21]. Additional oomycete *Avr* genes that do not encode RXLR effectors have also been proposed, such as *Avr3b-Avr10-Avr11* in *P. infestans* [24], [25] and *Avr1b*-2 in *P. sojae* [6]. Likewise, many new *Avr* genes from fungal plant pathogens are now being identified or proposed [4].

Our finding that the *Avr3c* gene of *P. sojae* lies in close proximity to the *Avr4/6* gene substantiates past studies describing genetic linkage of these avirulence determinants [22]. The clustering of *Avr* genes in *P. sojae* and *P. infestans* was discovered by studying F_2_ populations and hybrids [26], [27], [28], [29]. With the completion of whole genome sequences and associated physical maps, identification of one *Avr* gene may facilitate the identification of additional *Avr* genes that are genetically linked, as we have demonstrated in this study.

Results from the co-bombardment analysis show that *Avr3c* is able to trigger cell death (measured by reduced GUS expression) specifically in plants carrying *Rps3c*. This occurred regardless of the presence of the signal peptide, as has been noted for other *Avr* genes [13], [16]. Previous studies have demonstrated that the RXLR motif is necessary and sufficient for delivery of proteins into plant cells [12], [13], and it is now widely accepted that the RXLR motif functions as a host targeting signal. Thus, expression of full-length Avr3c in plant cells likely results in the protein being exported via the secretory pathway and re-imported via the RXLR mediated pathway. Overall, the results suggest that *Avr3c* is recognized within the plant cell.

As more *Avr* genes are identified in *P. sojae*, it is possible to compare their expression patterns to one another and to other RXLR effectors in the genome. Our results in this area show that RXLR effectors have varying patterns of expression, but most are expressed at their highest levels during early infection and in germinating zoospores. These results are not surprising because there is evidence that RXLR effectors assist the colonization of plant tissues by suppression of host defense responses [21], [30]. This will be especially crucial during the early stages of colonization, while *P. sojae* is growing biotrophically on the host. Previous work has shown that by 48 h after inoculation, *P. sojae* transits to a necrotrophic growth mode that is accompanied by expression of hydrolytic enzymes and toxins [31], [32], [33], [34]. Despite the overall similarity of RXLR effector expression, we noted differences and found groups that shared distinct expression patterns. For example, the expression of *Avr3c* and *Avr1b*-1 abruptly decline at 48 h after infection, while *Avr3a* and many other effectors continue to be expressed well into this necrotropic phase. These expression differences may be related to varying functional roles that the effectors engage in with the host, and the need for the pathogen to coordinate effector gene expression with that of particular host targets.

The arrangement of the *Avr3c* locus provides yet another example of a multi-copy *P. sojae Avr* gene occurring in a tandem array of duplicated DNA segments. Recent studies have shown that *P. sojae Avr1a* and *Avr3a* are multi-copy genes that display copy number variation among different *P. sojae* strains [16]. Although we noted sequence polymorphisms within the *Avr3c* and *Avh27b* genes among different *P. sojae* strains, there was no evidence for copy number variation of the repetitive unit among strains, as described for *Avr1a* and *Avr3a*. The *P. sojae* strains that are virulent on *Rps3c* plants display amino acid polymorphisms in the effector domain of Avr3c. Evasion of immunity by amino acid substitutions within the effector domain has also been described for *P. sojae* Avr1b-1 [6], *P. infestans* Avr3a [9], and *H. arabidopsis* ATR13 [7] and ATR1 [8]; whereas premature stop codons cause truncated proteins and gain of virulence at the *P. infestans Avr4* locus [17]. Transcriptional variation represents another mechanism for gain of virulence, as described for *P. sojae Avr1b*-1, *Avr1a*, and *Avr3a* [6], [16].

The arrangement of *Avr* genes in clusters offers opportunities for variation generated by sequence exchanges via unequal crossing over, homologous recombination, or gene conversion. Our results suggest that such processes occurred at the *P. sojae Avr3c* locus. Gain of virulence on *Rps3c* plants requires mutations to each of the two identical copies of *Avr3c^P6497^* that occur in avirulent strains of *P. sojae*. The *P. sojae* strains P7064 and ACR6 are virulent on *Rps3c* and carry two identical copies of the *Avr3c^P7064^* allele. The mutations that define the *Avr3c^P7064^* sequence could not have simultaneously arisen in the two *Avr3c* gene copies. Rather, these changes must have occurred in one copy and then spread to the second copy by sequence exchanges. It is also evident that four *P. sojae* strains, ACR8, ACR9, ACR17 and ACR24, have further evolved at this locus, by accumulating additional mutations in the *Avr3c^ACR9^* and *Avr3c^ACR8^* alleles. In these four *P. sojae* strains, the *Avr3c^ACR9^* and *Avr3c^ACR8^* alleles are clearly derived from *Avr3c^P7064^* but no sequence homogenization has occurred between the two copies of *Avr3c*.

The finding that *Avr3c* is a multi-copy RXLR effector that acquired gain of virulence mutations that spread through sequence exchanges provides a new example of the plasticity of the RXLR effector family. Similar mechanisms for generating diversity at plant resistance (*R*) gene loci have long been known [35], [36]. Thus, plant immune systems and pathogen effector systems mirror each other in an additional way, by relying on gene clusters and associated sequence exchange mechanisms to provide rapid and novel changes to genes that control immunity and virulence.

## Materials and Methods

### *Phytophthora sojae* isolates, plant materials and disease assays

Working stocks of *P. sojae* was routinely grown on 9cm 26% (v/v) V8 agar plates at 25°C for 5 to7 days in the dark [37]. The source of *P. sojae* isolates used in this study is described in Table S2. For plant infection assays *P. sojae* cultures were grown on V8 media containing 0.9% agar. Axenic cultures for nucleic acid isolation were prepared by transferring 5 mm mycelial disks cut from the growing edge of each colony to vegetable juice (V8) agar plates layered with a disc of cellophane (BioRad). After growth, cellophane sheets overlaid with fully grown mycelial colonies were peeled off the media and flash frozen in liquid nitrogen for extraction.

Soybean (*Glycine max*) cultivar Williams (*rps3c*) and the Williams isoline L92-7857 (*Rps3c*), were from the collection at Agriculture and Agri-Food Canada (Harrow, Ontario) and used to evaluate the virulence of *P. sojae* cultures. Etiolated soybean seedlings were grown in vermiculite soaked in 3 mg/L fertilizer (15-30-15) at 25°C day (16 h) and 16°C night (8 h) temperatures for 7 days prior to harvest for disease assays. A mycelial plug (5 mm diameter), cut from the growing edge of 5 to 7 day old V8 grown *P. sojae* cultures, was transferred to each of 15 to 20 hypocotyls per cultivar, mycelial-side down, 2 to 3 cm from the base of the cotyledon. For light-grown soybeans, six soybean seeds were sown in 10 cm pots (a minimum of three pots per isolate) containing soil-less mix (Pro-Mix ‘BX’, Premier Horticulture Ltd, Rivière-du-Loup, Canada) soaked with 3 mg/L fertilizer (20-20-20). Plants were grown in a controlled growth chamber with a 16 h photoperiod, 25°C day and 16°C night temperatures. Plants were grown for one week for use in virulence assays, or for two weeks for use in biolistics. *P. sojae* cultures were grown on 0.9% (v/v) V8 agar plates 5 to 7 days prior to light-grown plant inoculations. Mycelia inoculums were prepared by passing the actively growing edge of a culture through a 3 ml syringe attached to an 18-gauge needle. Soybean plants were inoculated in the mid-section of each hypocotyl by making a small incision for injection of the mycelial slurry. Inoculated plants were covered with plastic bags to maintain humidity for two days. Disease symptoms were allowed to develop for an additional four days before phenotypes were scored as resistant, susceptible or intermediate. A minimum of three independent replicates of the disease assay were performed for each *P. sojae* culture tested.

### Microarray hybridization and analysis

*P. sojae* P6497 and the compatible soybean cultivar Harosoy, were used for all treatments. Methods for isolation of mRNA from germinating zoospores, infected plant tissues, and mycelia have been described [31], [33]. Plant inoculations were performed by placing mycelia plugs on etiolated hypocotyls. A minimum of three biological replicates were performed for each of the seven treatments in the microarray experiment. Integrity, purity and concentration of RNA were verified by electrophoresis (Bioanalyzer, Agilent Technologies) before hybridization to high-density oligonucleotide arrays containing probe-sets for 15,820 predicted *P. sojae* genes (Affymetrix Soybean GeneChip). Data was normalized and analyzed using computer software (GeneSpring GX 7.3). Spot intensity from was interpreted through an RMA pre-processor. The following normalization steps were sequentially performed: (1) Data transformation: set measurements less than 0.01 to 0.01; (2) Per chip: normalize to a set of pre-determined genes, using all 15,820 *P. sojae* genes as control genes; (3) Per gene: normalize to median. To obtain a set of RXLR genes on the array, the nucleotide sequences of 385 predicted *P. sojae Avh* genes were downloaded from JGI *P. sojae* genome assembly1.1. To determine the *Avh* genes with corresponding probes on the microarray, computer software was used (www.affymetrix.com/analysis/netaffx/index.affx). Predicted *Avh* genes with sequences exactly matching to 11/11 probe-sets were considered as having a perfect probes on the microarray.

### Plasmid construction and plant transient expression assays

Primers used in this study are provided in Table S3. The primer sets named Avh27gF/Avh27gR, Avh28gF/Avh28gR, and Avh26gF/Avh26gR were used to amplify the *Avh27*, *Avh28* and *Avh26*, respectively. For each amplification product, PCR bands were excised and purified (QIAquick Gel Extraction Kit, Qiagen) prior to sequencing and sub-cloning. Each of the *Avh27a* and *Avh27b* constructs, with or without signal peptide, were amplified by using different combinations of primers. Products were digested with *Bgl*II and *Sph*I and inserted into the PFF19 plant transient expression vector. Recombinant plasmids were purified for sequencing and for plant transient expression assays. Co-bombardment assays were performed as previously described [32], [38]. After bombardment, leaves were left at room temperature for 16 h then transferred to GUS staining solution and incubated in darkness at 37 C for 16 h [32], [38]. Leaves were then washed in 70% (v∶v) ethanol and photographed using a digital camera.

### Genotyping of F_2_ progeny

Fragments amplified by primer sets Avh27abF-SP/Avh27aR and A27bRT-F/A27bRT-R were digested by *Rsa*I and *Xmn*I, respectively. The assays provided co-dominant markers for scoring the genotype of *P. sojae* P6497 or P7064 alleles of *Avh27a* and *Avh27b*. The F_2_ populations resulting from a cross of *P. sojae* P6497×P7064 have been described [23], [27]. A total of 40 F_2_ individuals were selected, based upon their genotypes for *Avh27a* and *Avh27b*, and their virulence was assessed on *Rps3c* soybean plants.

### Nucleic acid isolation and copy number determination

Methods for RNA isolation and reverse transcriptase PCR from *P. sojae* mycelium, zoospores, germinating zoospores, and *P.sojae*-infected soybean tissues have been described [31], [33]. Genomic DNA was isolated from *P. sojae* mycelial cultures using a modified CTAB (hexadecyl trimethyl ammonium bromide) method [39]. Gene copy number determinations were made by quantitative PCR, using an instrument that measures products in real-time (LightCycler, Roche, Laval, PQ, Canada, software version 3.5). A reference plasmid construct containing the *P. sojae* genes *CL164* and *Avr3c* was used to develop a standard curve with data points ranging from 1 to 108 copies. The gene *CL164* is present in single copy in the genome of *P. sojae* P6497. Primers specific for *Avr3c* and *CL164* are available in Table S3. Amplification reactions (20 µl) were performed in duplicate with 106.6 or 1066 pg of input genomic DNA, 3.25 mM MgCl_2_, 0.5 µM of each primer and 2 µl of master mix (FastStart DNA Master SYBR Green I mix, Roche). The PCR parameters were as follows: an initial 10 min denaturation step at 95°C followed by 40 cycles of 15 s at 95°C, 10 s at 65°C and 12 s at 72°C. Specificity of the primers was verified by a melting curve analysis of the PCR products with a temperature gradient of 0.2°C/s from 68°C to 98°C and by conventional gel electrophoresis. Copy number of *Avr3c* was determined as a ratio of the estimated copies of *Avr3c* to that of the reference gene, *CL164*. The estimation of gene copy number by counting of trace file matches from whole genome shotgun sequences was performed as previously described [16].

### Genome structure analysis

The 80 kb of genomic sequence encompassing *Avh27a* and *Avh27b* was downloaded from *P. sojae* genome assembly v3.0. Computer software (DNA Star, Lasergene) was used to search for imbedded repetitive segments. A replicate unit containing *Avh27b* was well-assembled, while another nearly identical unit containing *Avh27a* contained a gap in the assembly. After analysis and re-assembly, it was determined that two copies of the replicate unit containing *Avh27a* were present in the genome of *P. sojae* P6497. The re-assembled *Avh27* genomic region was compared to syntenic regions in *P. ramorum* (JGI *P. ramorum* v1.1 database), *P. infestans* (Broad Institute, *Phytophthora* Database) and *Hyaloperonospora arabidopsis* (VBI microbial database, *H. arabidopsis* assembly v3.0).

### Data Deposition

The sequence data for the *Avr3c* alleles have been deposited to NCBI GenBank under the accession numbers: FJ705360, FJ705361, FJ705362 and FJ705363. Microarray expression data has been deposited to NCBI-GEO, series GSE15100.

## Supporting Information

Figure S1 Conservation and interruption of synteny of the *Avr3c* region in *P. sojae*, *P. ramorum*, *P. infestans*, and *Hyaloperonospora arabidopsis*. (A) Comparison of the *Avr3c* region in four different oomycete species. Colored block arrows indicate the position and transcriptional orientation of putative open reading frames (ORF). Orthologous genes, indicating conservation of synteny, are linked by dashed lines. Paralogous genes are shown in the same color. The RXLR effector genes *Avr3c* and *Avh27b* are shown in red.
(0.38 MB TIF)

Table S1 Predicted Open Reading Frames (ORF) in the 33.7 kb replicate unit.
(0.05 MB DOC)

Table S2 List of *Phytophthora sojae* strains used in this study
(0.06 MB DOC)

Table S3 List of primers and probes used in this study of the *P. sojae Avr3c* locus
(0.07 MB DOC)
