# Supplementary figures and images for: The Phytophthora sojae Avirulence Locus Avr3c Encodes a Multi-Copy RXLR Effector with Sequence Polymorphisms among Pathogen Strains
Source: PLoS One. 2009 May 15;4(5):e5556. doi: 10.1371/journal.pone.0005556 (PMC2678259; doi:10.1371/journal.pone.0005556)

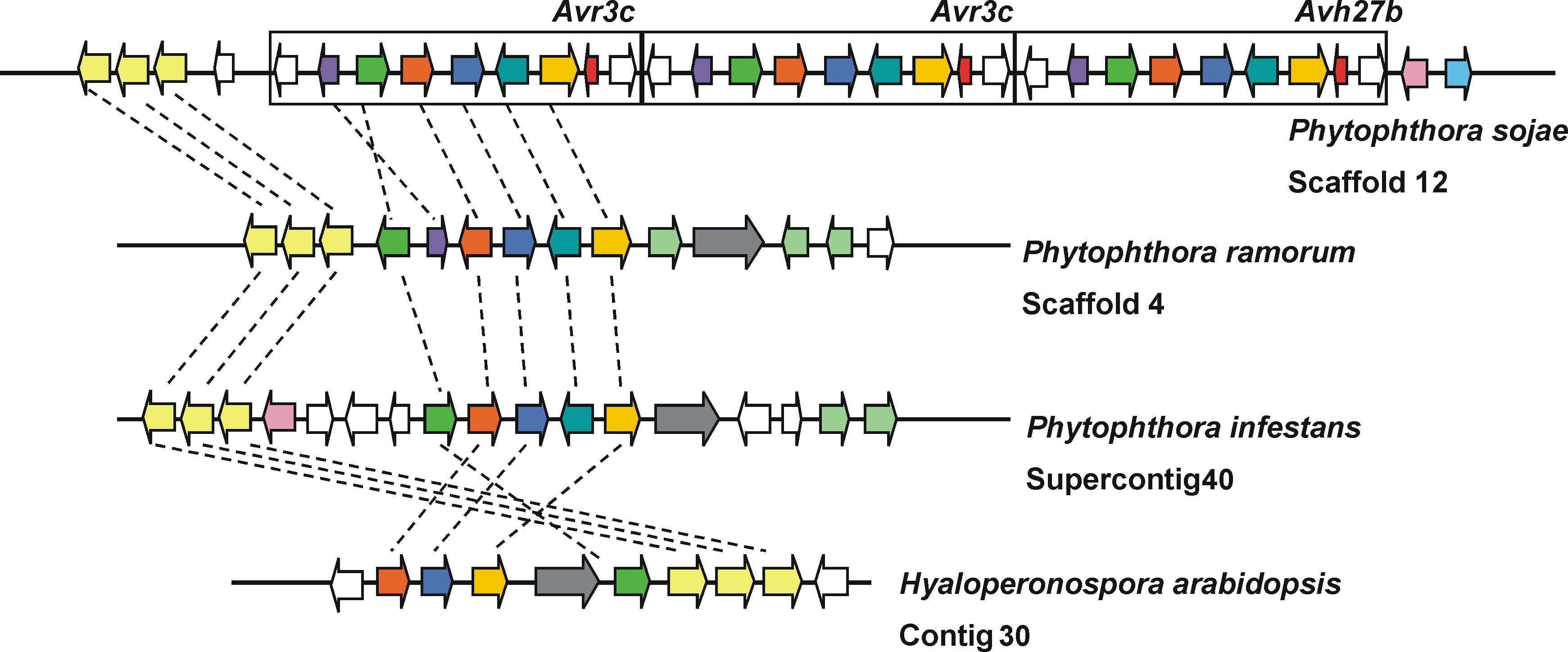

Supplement: Figure S1 — Conservation and interruption of synteny of the Avr3c region in P. sojae, P. ramorum, P. infestans, and Hyaloperonospora arabidopsis. (A) Comparison of the Avr3c region in four different oomycete species. Colored block arrows indicate the position and transcriptional orientation of putative open reading frames (ORF). Orthologous genes, indicating conservation of synteny, are linked by dashed lines. Paralogous genes are shown in the same color. The RXLR effector genes Avr3c and Avh27b are shown in red. (0.38 MB TIF) [file pone.0005556.s001.tif]
