# Supplementary material for: The Phytophthora sojae Avirulence Locus Avr3c Encodes a Multi-Copy RXLR Effector with Sequence Polymorphisms among Pathogen Strains
Source: PLoS One. 2009 May 15;4(5):e5556. doi: 10.1371/journal.pone.0005556 (PMC2678259; doi:10.1371/journal.pone.0005556)
Supplement: Table S1 — Predicted Open Reading Frames (ORF) in the 33.7 kb replicate unit. (0.05 MB DOC) [file pone.0005556.s002.doc]

| **Table S1.** Predicted Open Reading Frames (ORF) in the 33.7 kb replicate unit. | | | | | |
| --- | --- | --- | --- | --- | --- |
| Name | Gene ID ^a^ | EST^b^ | Length ^c^ | BLAST result (E<10^-5^) ^d^ | E-value^e^ |
| ORF1 | 130712 |  | 136 | - | - |
| ORF2 | 130711 | CL2225Contig1 | 237 | - | - |
| ORF3 | 130710 |  | 502 | NOL1/NOP2/sun family tRNA and rRNA cytosine-C5-methylases  [*Arabidopsis thaliana*] | 10^-68^ |
| ORF4 | 108501 | CL1116Contig1 | 368 | metal-dependent protein hydrolase  [*Dictyostelium discoideum*] | 10^-81^ |
| ORF5 |  | psZO004iB11r | 256 | - | - |
| ORF6 | 156203 |  | 176 | - | - |
| ORF7 | 156202 |  | 208 | methylase  [*Tetraodon nigroviridis*] | 10^-47^ |
| Avh27 |  |  | 221/231 | - | - |
| ORF8 | 130705 | CL519Contig1 | 351 | - | - |
| ^a^Nine ORF including those encoding Avh27a or Avh27b were predicted to occur in the 33.7 kb replicate unit. Seven of the ORF had gene models from the *P. sojae* genome assembly v1.1, as indicated.  ^b^Four ORF had matching Expressed Sequence Tag (EST) sequences.  ^c^The length of the predicted ORF; number of codons including stop codon.  ^d^The translated amino acid sequences were compared to known protein sequences in NCBI databases by BLAST analysis, using a cut-off Expect (E) value of 10^-5^.  ^e^Expect value returned from BLAST analysis. | | | | | |
