# Supplementary material for: The Phytophthora sojae Avirulence Locus Avr3c Encodes a Multi-Copy RXLR Effector with Sequence Polymorphisms among Pathogen Strains
Source: PLoS One. 2009 May 15;4(5):e5556. doi: 10.1371/journal.pone.0005556 (PMC2678259; doi:10.1371/journal.pone.0005556)
Supplement: Table S2 — List of Phytophthora sojae strains used in this study (0.06 MB DOC) [file pone.0005556.s003.doc]

| **Table S2.** List of *Phytophthora sojae* strains used in this study | | | | |
| --- | --- | --- | --- | --- |
| Name | Strain | Virulence^a^  *Rps3c* | Origin^b^ | From^c^ |
| race 1 | 48FPA18 | A | Ohio (F.S.) | F.S. |
| race 2 | P6497 | A | Mississippi (B.K.) | B.M.T. |
| race 3 | 25MEX4 | A | Ohio (F.S.) | F.S. |
| race 6 | ACR6 | V | London, ON (E.W.) | T.A. |
| race 7 | P7064 | V | Canada (C.M.) | B.M.T. |
| race 8 | ACR8 | V | Unknown | T.A. |
| race 9 | ACR9 | V | Harrow, ON (C.M.) | T.A. |
| race 10 | ACR10 | A | Stoneville, MS (B.K.) | T.A. |
| race 11 | ACR11 | A | Harrow, ON (C.M.) | T.A. |
| race 12 | ACR12 | A | Stoneville, MS (B.K.) | T.A. |
| race 16 | ACR16 | A | Stoneville, MS (B.K.) | T.A. |
| race 17 | ACR17 | V | Stoneville, MS (B.K.) | T.A. |
| race 17 | P7074 | V | Stoneville, MS (B.K.) | B.M.T. |
| race 19 | P7076 | A | Stoneville, MS (B.K.) | B.M.T. |
| race 20 | ACR20 | A | Unknown | T.A. |
| race 21 | ACR21 | A | Lafayette, IN (F.A.L) | T.A. |
| race 24 | ACR24 | V | Lafayette, IN (F.A.L) | T.A. |
| race 25 | ACR25 | A | Lafayette, IN (F.A.L) | T.A. |
| ^a^Virulence on soybean *Rps3c* gene; A, avirulent; V, virulent.  ^b^Original site of isolation and where known, investigator who conducted the isolation.  ^c^Investigator who provided the culture to Agriculture and Agri-Food Canada, London, ON. T.A. = T. Anderson (Agriculture and Agri-Food Canada, Harrow, ON); B.K.= B. Keeling (USDA, Stoneville, MS); F.A.L.= F.A. Laviolette (Purdue University, West Lafayette, IN); C.M = C. Meharg (Agriculture and Agri-Food Canada, Harrow, ON); F.S.= F. Schmitthenner (Ohio State University, Wooster, OH); B.M.T.= B. M. Tyler, (Virginia Bioinformatics Institute, Blacksburg, VI); E.W. = Ed Ward (Agriculture and Agri-Food Canada, London, ON). | | | | |
