# Supplementary material for: The Phytophthora sojae Avirulence Locus Avr3c Encodes a Multi-Copy RXLR Effector with Sequence Polymorphisms among Pathogen Strains
Source: PLoS One. 2009 May 15;4(5):e5556. doi: 10.1371/journal.pone.0005556 (PMC2678259; doi:10.1371/journal.pone.0005556)
Supplement: Table S3 — List of primers and probes used in this study of the P. sojae Avr3c locus (0.07 MB DOC) [file pone.0005556.s004.doc]

| **Table S3.** List of primers and probes used in this study of the *Phytophthora sojae Avr3c* locus | | |
| --- | --- | --- |
| **Name** | **Sequence (5'-3')** | **Use** |
| Avh27abF+SP (Bgl II) | ACTAGATCTATGCGCGTGTGCTCCGTC | Forward primers for *Avh27a* and *Avh27b* (with signal peptide) |
| Avh27abF-SP (Bgl II) | ACTAGATCTATGGTTGCTGCAGCTGCTCTCA | Forward primers for *Avh27a* and *Avh27b* (without signal peptide) |
| Avh27bR (Sph I) | ACTGCATGCTTACATGAGTCGCCGTAGGAA | Reverse primers for *Avh27b* |
| Avh27aR (Sph I) | ACTGCATGCTTACTTGTGTTTCCTTCGGTAT | Reverse primers for *Avh27a* (allele P6497 and P7064) |
| Avh27aR-2 (Sph I) | ACTGCATGCTTACTTGTGTTTCATTCGGTAT | Reverse primers for *Avh27a* (allele ACR9 and ACR8) |
| Avh27aRT-F | TCAAAAAGTGGATAGAAGAAAAAC | To determine the transcriptional pattern of *Avh27a* |
| Avh27aRT-R | ACCCACGCTTTGTTTAGTCTCT |
| Avh27bRT-F | TGGTTGAATGGCCCGCCTGA | To determine the transcriptional pattern of *Avh27b* |
| Avh27bRT-R | GATCCCCTTTCCATATTCGTG |
| Avh28RT-F | CTAATTGCTGCAGCCACTCTC | To determine the transcriptional pattern of *Avh28* |
| Avh28RT-R | TCGCTCAACCGGACAAGTG |
| Avh26RT-F | CGACGCGGTTAGTGAGAACT | To determine the transcriptional pattern of *Avh26* |
| Avh26RT-R | TCGAGCCTGCATAACGTCTG |
| PrbAvh27ab_F | AAGCCCTTTCAAGTCCTCAGTG | To produce probe designed for *Avh27a* and *Avh27b* conserved region, for DNA blot analysis |
| PrbAvh27ab_R | TACCGTCTTCTGCCTCGTTGA |
| ProAvh27b | CTCCCCAACCTGTCGCCTGTAGACGACGCACTC  TCCGGGCTCAAGAACGCcGTGAAGATCAGCCCT | Probe and primers designed for *Avh27b*-specific region, for DNA blot analysis |
| ProAvh27b_F | ACTCCCCAACC |
| ProAvh27b_R | TCAGGGCTGATC |
| ClAvh27F | AATGCATGCCACCGTAGATCTTCGCCTGCT | To clone *Avh27a* into vector |
| ClAvh27R | AATGCATGCACCCACGCTTTGTTTAGTCTCT |
| Real-abF | CAAGCCCTTTCAAGTCCTCAG | To determine copy number of *Avh27a* and *Avh27b* |
| Real-abR | TACCGTCTTCTGCCTCGTTGA |
| Real-aF | AGTGAAGAGAGAGGACTATTCG | To determine copy number of *Avh27a* |
| Real-aR | CCATTGGCGGTTCTTTTATCG |
| Real-CLF | GAGGGCATGTTCGCGGAGAT | For use as a single copy control gene in real-time PCR |
| Real-CLR | GTCGGCTCCTCGTACTTCT |
| Avh27gF | TGAACCTTCCAGTGCAGTAC | To sequence *Avh27* polymorphism |
| Avh27gR | AGCCCTGAATCTGACCTCTT |
| Avh28gF | CGTGCATGTACTAATCGATAAA | To sequence *Avh28* polymorphism |
| Avh28gR | CGACAACATGGACGGCAGA |
| Avh26gF | ATACATGTAGCTCGTGAATAGA | To sequence *Avh26* polymorphism |
| Avh26gR | GGAGAAGAAGACCTGCAGAC |
